# Nitric oxide produced by NOS2 copes with the cytotoxic effects of superoxide in macrophages

**DOI:** 10.1016/j.bbrep.2021.100942

**Published:** 2021-02-20

**Authors:** Sho Kobayashi, Takujiro Homma, Junichi Fujii

**Affiliations:** Department of Biochemistry and Molecular Biology, Graduate School of Medical Science, Yamagata University, 2-2-2 Iidanishi, Yamagata City, Yamagata, 990-9585, Japan

**Keywords:** Macrophage, Nitric oxide, Superoxide, NOS2, SOD1

## Abstract

Nitric oxide (NO) reacts with superoxide to produce peroxynitrite, a potent oxidant and reportedly exerts cytotoxic action. Herein we validated the hypothesis that interaction of NO with superoxide exerts protection against superoxide toxicity using macrophages from mice with a knockout (KO) of inducible NO synthase (NOS2) and superoxide dismutase 1 (SOD1), either individually or both. While no difference was observed in viability between wild-type (WT) and NOS2KO macrophages, SOD1KO and SOD1-and NOS2-double knockout (DKO) macrophages were clearly vulnerable and cell death was observed within four days. A lipopolysaccharide (LPS) treatment induced the formation of NOS2, which resulted in NO production in WT and these levels were even higher in SOD1KO macrophages. The viability of the DKO macrophages but not SOD1KO macrophages were decreased by the LPS treatment. Supplementation of NOC18, a NO donor, improved the viability of SOD1KO and DKO macrophages both with and without the LPS treatment. The NOS2 inhibitor nitro-l-arginine methyl ester consistently decreased the viability of LPS-treated SOD1KO macrophages but not WT macrophages. Thus, in spite of the consequent production of peroxynitrite in LPS-stimulated macrophages, the coordinated elevation of NO appears to exert anti-oxidative affects by coping with superoxide cytotoxicity upon conditions of inflammatory stimuli.

## Highlights

• SOD1 deficiency decreases the life span of primary cultured macrophages.
• A NO donor prolongs the survival of SOD1-deficient macrophages.
• NOS2 inhibition shortens the life span of SOD1-deficient macrophages.
• NO reduces superoxide cytotoxicity under conditions of an SOD1 deficiency.

## Abbreviations

NO: nitric oxide
NOS2: inducible nitric oxide synthase
SOD: superoxide dismutase
KO: knockout
WT: wild type
DKO: SOD1 and NOS2 double knockout
LPS: lipopolysaccharide
NOX2: NADPH oxidase 2
LDH: lactate dehydrogenase
l-NAME: nitro-l-arginine methyl ester
COX2: cyclooxygenase-2
ROS: reactive oxygen species
H_2_DCF-DA: 2′,7′–dichlorofluorescein diacetate
GSH: glutathione
Gpx1: glutathione peroxidase 1
Prx: peroxiredoxin
LC-MS: liquid chromatography-mass spectrometry
NEM: N-ethylmaleimide
CAT: cationic amino acid transporter

## 1 Introduction

Inflammatory stimuli, such as a lipopolysaccharide (LPS) and interferon-γ, induce the expression of nitric oxide synthase 2 (NOS2) and trigger the production of a large amount of nitric oxide (NO) [1]. It has been reported that NO exerts bactericidal and tumoricidal effects, as evidenced by the finding that NOS2 knockout (KO) mice fail to inhibit the replication of *Listeria monocytogenes* or lymphoma cells [2]. Another study reported that NO functions as a host defense in *Salmonella* infections via exerting a direct antimicrobial effect and, at the same time, exerting cytoprotective actions for infected host cells [3].

Activated macrophages also produce large amounts of reactive oxygen species (ROS) [4]. NADPH oxidase 2 (NOX2) catalyzes the production of superoxide radicals via an electron transfer from NADPH to an oxygen molecule and contributes to the bactericidal action [5]. Excessive levels of superoxide, however, may also damage the host tissues. Superoxide dismutase (SOD) functions to decrease superoxide to allowable levels in cells [6], and hence a deficiency of SOD leads to the development of a variety of oxidative stress-related pathological conditions [7]. Among the three SOD isozymes, SOD1 is present abundantly in cytoplasm and in the intermembrane space of mitochondria [8]. Because the levels of oxygen under cell culture conditions are about 4-times higher than the arterial blood level in vivo, oxidative damage is more pronounced. For example, while SOD1KO mouse embryonic fibroblasts die within 24 h in conventional cultures, they can survive under conditions of hypoxic culture [9].

NO interacts with ROS and is converted into several reactive nitrogen oxide species [10]. The interaction of NO with superoxide is faster than the dismutation reaction catalyzed by SOD and results in the formation of peroxynitrite (ONOO^−^), which exerts strong bactericidal action [11]. Due to its high reactivity, macrophages themselves may also be oxidatively damaged by peroxynitrite, notably under conditions of a SOD1 deficiency [12]. For example, it has been reported that peroxynitrite causes accelerated muscle loss in SOD1KO mice [13]. In the case of the vascular system, however, peroxynitrite appears to play a signaling role in prostanoid synthesis [14]. Thus, the issue of how peroxynitrite acts, either harmful or beneficial, may also depend on type of cells involved and the marginal environment.

We report herein on attempts to gain insights into the issue by using peritoneal macrophages from mice with a deficiency in the NOS2 and/or SOD1 genes. The resulting findings might be contrary to the current general understanding of the biological consequence of interaction between nitric oxide and superoxide.

## 2 Materials and methods

### 2.1 Materials

All chemicals and agents were purchased from Sigma-Aldrich (St. Louis, MO, USA) or Fujifilm Wako Pure Chemicals Co. (Osaka, Japan), unless otherwise stated.

### 2.2 Mice

Sod1^−/−^ (SOD1KO) mice, which were originally established by Matzuk et al. [15], were purchased through the Jackson Laboratories and backcrossed more than 10 times with WT C57BL/6 N mice at our institute [16]. NOS2^−/−^ (NOS2KO) mice, which were originally established by Laubach et al. [17] and purchased through Jackson Laboratories, were also bred in our institution. Sod1^−/−^;NOS2^−/−^ (DKO) mice were established by mating male SOD1KO mice and female NOS2KO mice. The animal room was maintained under specific pathogen-free conditions at a constant temperature of 20–22 °C with a 12-hr alternating light-dark cycle. Animal experiments were performed in accordance with the Declaration of Helsinki under the protocol approved by the Animal Research Committee at our institution.

### 2.3 Isolation and culture of elicited peritoneal macrophages

Peritoneal macrophages were collected and cultured as described previously [18]. Briefly, mice were given an intraperitoneal injection of 2.0 mL of a 4.0% thioglycolate broth. Four days after this injection, macrophages were collected by peritoneal lavage. The cells were washed with serum-free RPMI 1640 medium and plated at 1.0 × 10^6^ cells/35-mm diameter culture dish in complete RPMI 1640 medium (RPMI 1640, 10% fetal bovine serum, 100 units/mL penicillin, and 100 μg/mL streptomycin). At 2.0 h after plating, the medium was replaced to remove non-adherent cells and the remaining cells were exposed a 1.0 μg/ml solution of bacterial LPS (L6386, Sigma Aldrich) for the indicated times. Where indicated, the cells were treated with l-NAME (sc-200333, Santa Cruz Biotechnology, Santa Cruz, CA, USA) or NOC18 (82120, Cayman Chemical, Ann Arbor, MI, USA).

### 2.4 Assay of cell viability

Cellular viability was assessed under light-microscopy. Briefly, images of the cells were obtained at the indicated time points and intact cells adhering to the dish were counted manually. Fragmented, condensed, and shrunken cells were regarded as dead or dying cells. The percentage of living cells was determined by dividing the number of cells at each time point by the initial cell number (0 h) as 100%.

### 2.5 Assay of cytotoxicity

Cytotoxicity was determined by means of a lactate dehydrogenase (LDH) assay. The reaction mixture contained 20 μl of culture medium, 0.3 mM NADH, 1.0 mM sodium pyruvate, and 200 mM sodium phosphate buffer, pH 7.2 in a total of 100 μl. Initial activities were calculated from the rate of disappearance of NADH during the starting linear phase of the reaction by monitoring the absorbance at 340 nm using Varioskan Flash (Thermo Fisher Scientific, Yokohama, Japan).

### 2.6 Determination of nitrite levels

The levels of nitrites, oxidized metabolites derived from NO, were assessed by means of the Griess reaction as described [18]. The content of nitrites in the sample was determined using NaNO_2_ as the standard.

### 2.7 Protein preparation

Cells were lysed in Ripa buffer, which contained 25 mM Tris-HCl, pH 7.5, 150 mM NaCl, 1.0% (w/v) Nonidet P-40, 1.0% (w/v) sodium deoxycholate, 0.1% (w/v) SDS with a protease inhibitor cocktail (Sigma Aldrich). After centrifugation at 22,000×*g* in a microcentrifuge, protein concentrations were determined using a Pierce® BCA™ Protein Assay Kit (Thermo Fisher Scientific).

### 2.8 Western blotting

Aliquots of protein (20 μg) were separated on 6.0% or 15% SDS-polyacrylamide gels and electroblotted onto polyvinylidene difluoride (PVDF) membranes (Millipore, Tokyo, Japan). The blots were blocked by treatment with 5.0% skimmed milk in Tris-buffered saline containing 0.1% Tween-20, and were then incubated with antibodies. The following antibodies were used: SOD1 [16], SOD2 [16], NOS2 (sc-8310, Santa Cruz Biotechnology), cyclooxygenase-2 (COX2) (sc-1746, Santa Cruz Biotechnology), peroxiredoxin (Prx) 1 (Prx1) [19], Prx2 (LF-PA0091, AbFrontier, Seoul, Korea), Prx3 (LFMA0044, AbFrontier), Prx4 [20], glutathione peroxidase 1 (Gpx1) [21] and glyceraldehyde-3-phosphate dehydrogenase (GAPDH) (sc-25778, Santa Cruz Biotechnology). After incubation with horseradish peroxidase-conjugated anti-mouse (sc-2005, Santa Cruz Biotechnology) or anti-rabbit (sc-2004, Santa Cruz Biotechnology) secondary antibodies, the immunoreactive bands were detected using an Immobilon western chemiluminescent HRP substrate (Millipore) on an image analyzer (ImageQuant LAS500, GE Healthcare, Hino, Japan). The relative amounts of each protein were quantified using the Image J software [22].

### 2.9 Quantification of amino acids and glutathione

LC-MS analyses of the intracellular content of amino acids and GSH-related peptides were performed as described previously [23]. System control, data acquisition, and quantitative analysis were performed with the Xcalibur 2.2 software (Thermo Fisher Scientific). Standard curves for amino acids, GSH-NEM, and cysteine-NEM showed linearity in the concentration ranges examined.

### 2.10 Analysis of intracellular ROS by flow cytometry

Cells were incubated with 20 μM 2′,7′–dichlorofluorescein diacetate (H_2_DCF-DA, Thermo Fisher Scientific) in the culture medium for 15 min and then washed with phosphate-buffered saline. After trypsinization, the cells were collected and subjected to a FACS analysis (FACSCanto II, BD Biosciences, Tokyo, Japan) at an excitation wavelength of 488 nm and an emission wavelength of 517–527 nm.

### 2.11 Statistical analysis

Statistical analyses were performed using the GraphPad Prism 6 software (GraphPad, San Diego, CA, USA). Statistical significance was determined using two-way ANOVA followed by Tukey's post hoc test.

## 3 Results

### 3.1 SOD1 deficiency decreased viability of macrophages in culture

**Fig. 1:**
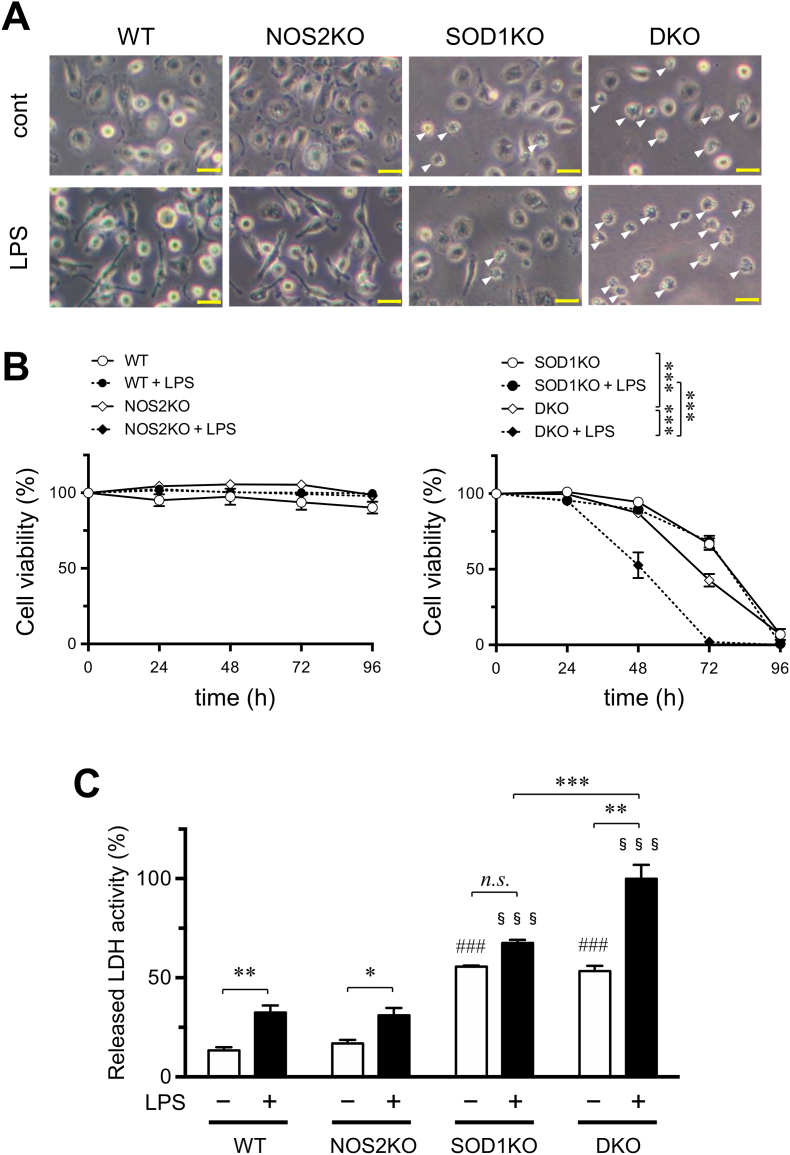
A deficiency of SOD1 and/or NOS2 affects viabilities in macrophages. Macrophages isolated from WT, NOS2KO, SOD1KO or DKO mice were cultivated for 2.0 h and then treated with or without LPS (1.0 μg/ml) at time 0 h. (A) Representative images of macrophages isolated from WT, NOS2KO, SOD1KO, or DKO mice. Isolated macrophages were cultivated for 72 h with or without LPS (1.0 μg/ml). Arrowheads indicate dead or dying cells that show fragmentation, condensation, or shrinkage. Scale bar, 20 μm. (B) Changes in the viability of WT, NOS2KO, SOD1KO or DKO macrophages. Data represent the mean ± SEM (n = 3–4). (C) Cytotoxicity of cells was assessed by measuring the LDH activity released into the culture media at 72 h. Relative values to those of LPS-treated DKO macrophages as 100%. Data represent the mean ± SEM (n = 3–4). *, P < 0.05; **, P < 0.01; ***, P < 0.001; ###, P < 0.001 vs WT LPS (−); §§§, P < 0.001 vs WT LPS (+). (Tukey's test) n.s., not significant.

Macrophages were isolated from WT, SOD1KO, NOS2KO and DKO mice and cultivated under conventional conditions. Cellular viability was assessed by counting cells under light-microscopy (Fig. 1A). The viabilities of WT and NOS2KO macrophages were unchanged during the incubation period, but the SOD1KO and DKO macrophages died within 4 days in culture (Fig. 1B). Because macrophages do not express NOS2 under control conditions, it is questionable why DKO macrophages were more vulnerable compared to the SOD1KO macrophage, but we do not have answer to this issue. LPS treatment induces the expression of NOS2 in macrophages [1]. To examine the issue of whether endogenously produced NO by NOS2 is protective in SOD1-deficient macrophages, we treated the macrophages with LPS and then stimulated them to induce NOS2 under cultivated conditions. When the macrophages were treated with LPS (1.0 μg/ml, final concentration) from 2.0 h after isolation and incubated further, a greater decrease in the viability of the DKO macrophages was detected, but no difference was found in the cases of the other genotypic macrophages. We then assessed cellular damage by measuring LDH activity that had been released from the destroyed macrophages. Activities of the medium LDH were increased by the LPS treatment, excluding that of the SOD1KO macrophages (Fig. 1C). Whereas some inconsistent results were obtained by the two methods that were used for assessing cellular viability and damage, the LPS treatment caused extensive damage to the macrophages, being the most severe of the treatments of the DKO macrophages.

### 3.2 NO production was elevated in WT and SOD1-deficient macrophages upon LPS stimulation

**Fig. 2:**
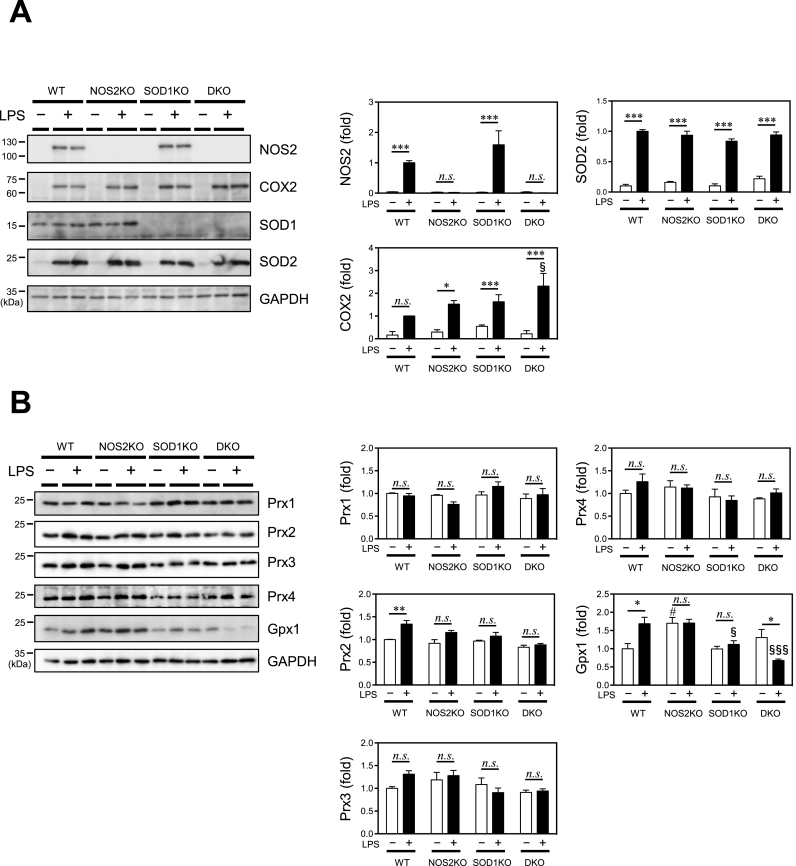
Levels of proteins in the macrophages. Proteins were extracted from macrophages that had been incubated with or without LPS (1.0 μg/ml) for 48 h and subjected to immunoblot analyses using the indicated primary antibodies. (**A**) Representative images and the quantification of the intensity of NOS2, COX2, SOD1, SOD2 are shown. Values relative to those of LPS-treated WT macrophages as 1.0. (**B**) Representative images and the quantification of the intensity of Prx1, Prx2, Prx3, Prx4 and Gpx1 proteins are shown. The quantification of the intensity are normalized to the corresponding GAPDH band. Values relative to those of LPS-untreated WT macrophages as 1.0. Columns and bars represent the mean ± SEM (n = 3). *, *P* < 0.05; **, *P* < 0.01; ***, *P* < 0.001; ^#^, *P* < 0.05 vs WT LPS (−); ^§^, *P* < 0.05 vs WT LPS (+); ^§§§^, *P* < 0.001 vs WT LPS (+). (Tukey's test) n.s., not significant.

We then examined the expression of enzymes responsible for the production of or the scavenging of NO and superoxide; NOS2, SOD1, SOD2 and COX2. NOX2 was not examined because post-translational modification by inflammatory stimuli is involved in this activation [5]. While the NOS2 protein was barely detected in cells that had been cultivated in conventional media, LPS stimulation induced NOS2 expression in WT and SOD1KO macrophages but not in NOS2KO or DKO macrophages (Fig. 2A). The COX2 protein was induced to differential extents in these cells after the LPS treatment but was not associated with viability of the cells. The SOD1 protein was constitutively present in WT and NOS2KO macrophages but was absent in SOD1KO and DKO macrophages, while the SOD2 protein was measurably induced by the LPS stimulation in all cells, as previously reported [24]. Prx2 and Gpx1 reportedly function as peroxynitrite reductases [25,26]. We therefore examined the protein levels of the Prx family, Prx1-4, and Gpx1 and found increases in levels of Prx2 and Gpx1 in WT macrophages by the LPS treatment but a decrease in the level of Gpx1 in DKO macrophages by the LPS treatment. Otherwise modest changes were observed in these proteins irrespective of the LPS treatment (Fig. 2B), suggesting that these proteins are not strongly associated with the viability of these cells.

**Fig. 3:**
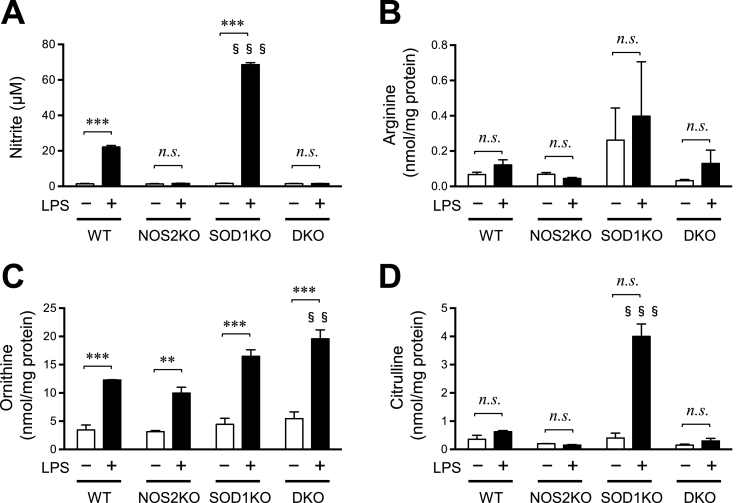
Levels of nitrite in cultured media and amino acids in the macrophages. (A) Nitrite in the culture medium was assayed by the Griess method. After measurement of amino acids and related compounds in the cells by LC-MS, values for (B) arginine, (C) ornithine, and (D) citrulline are shown. Values of other amino acids and glutathione, which showed significant differences among experimental groups, are available as Supplementary Table 1. Columns and bars represent the mean ± SEM (n = 3–4). **, P < 0.01; ***, P < 0.001; §§, P < 0.01 vs WT LPS (+); §§§, P < 0.001 vs WT LPS (+). (Tukey's test) n.s., not significant.

We then assessed the release of NO from macrophages by measuring nitrite, the oxidation product of NO in culture media. Nitrite levels were elevated only in the LPS-stimulated WT and SOD1KO macrophages but not in the NOS2-deficient cells (Fig. 3A). It is noteworthy that NO production was significantly higher in the SOD1KO macrophages than that for the WT macrophages upon LPS stimulation. The NOS2 protein catalyzes the production of NO from arginine and releases citrulline as another product [2]. Citrulline and ornithine as well as arginine are amino acids that are involved in the urea cycle, and hence their levels are correlated. We then measured contents of amino acids and GSH-related peptides, which are either associated with NO production or play a role on antioxidation, in these cells (Fig. 3B–D and Supplementary Table 1). Arginine, a substrate for the NOS2 reaction tended to be high in SOD1KO macrophages, especially upon LPS treatment (Fig. 3B). While the LPS treatment increased the ornithine content in all genotypic groups of macrophages, the level was higher in the macrophages with SOD1KO and DKO than the others (Fig. 3C). It is also interesting to note that the citrulline level was exceptionally high in SOD1KO macrophages with the LPS treatment (Fig. 3D).

### 3.3 DKO and SOD1KO macrophages produce higher levels of peroxides than WT and NOS2KO macrophages upon LPS stimulation

**Fig. 4:**
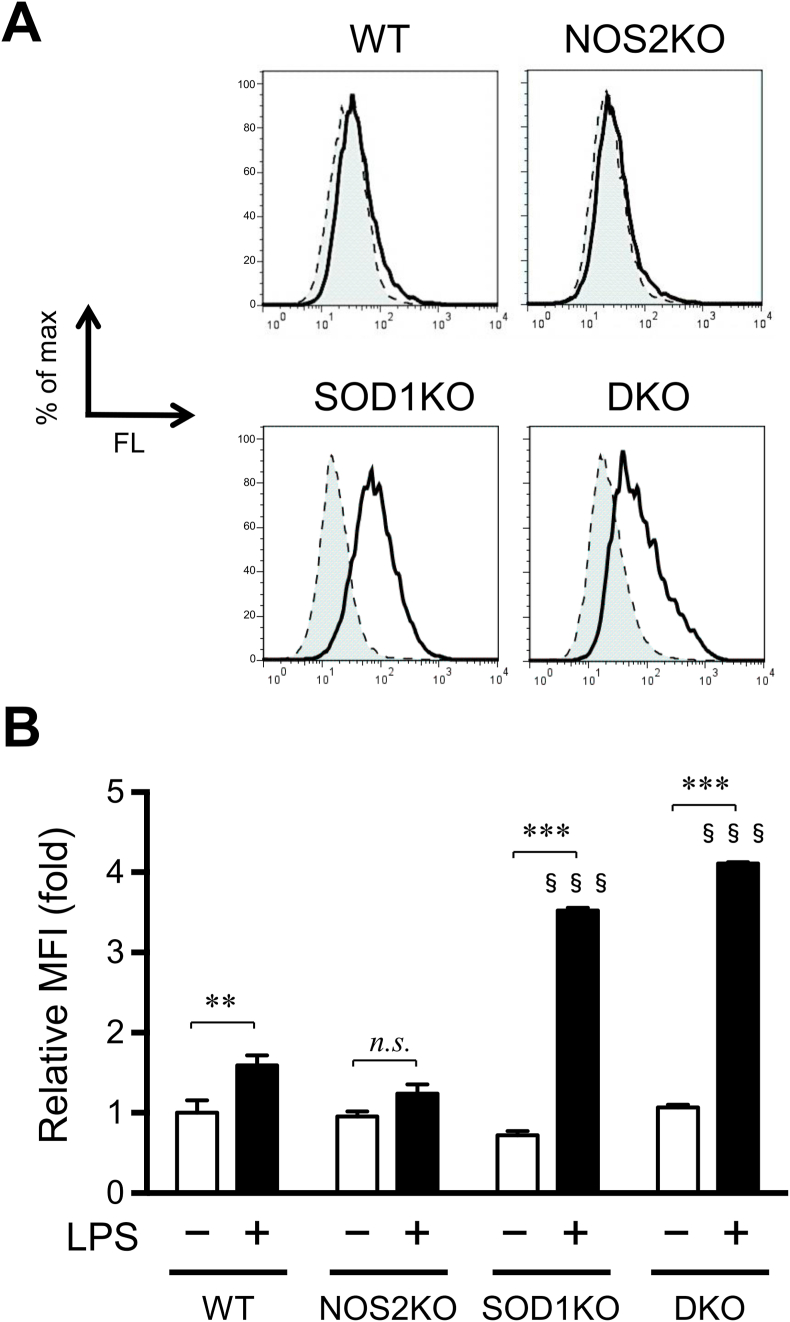
FACS analyses of ROS in macrophages. At 2 h after isolation, macrophages were treated with LPS (1.0 μg/ml). After 24 h cultivation with LPS, macrophages were incubated with a fluorescent probe 20 μM H_2_DCF-DA for 15 min, and then subjected to FACS analyses. (A) Representative charts that indicate changes of DCF fluorescence after LPS treatment are shown. Solid lines and dotted lines indicate LPS-treated and untreated cells, respectively. (B) Mean fluorescent intensity (MFI) of DCF are shown. Relative values to those of untreated WT macrophages as 1.0. Data represent the mean ± SEM (n = 4). **, P < 0.01; ***, P < 0.001; §§§, P < 0.001 vs WT LPS (+). (Tukey's test) n.s., not significant.

Superoxide is abundantly produced by activated NADPH oxidase in macrophages in response to LPS stimulation and is rapidly converted to hydrogen peroxide either spontaneously or via an SOD-catalyzed reaction sequence [6]. We assessed ROS levels in the macrophages using a peroxide-reactive fluorescent probe H_2_DCF-DA coupled with FACS analyses at 24 h after their isolation. No difference was observed in the ROS levels among the groups without LPS stimuli. While ROS levels were elevated only slightly in the WT and NOS2KO macrophages that had received the LPS treatment, their levels were markedly elevated in SOD1KO and DKO macrophages that had been treated with LPS (Fig. 4). LPS stimulation increased the fluorescent intensity to a greater extent in the DKO and SOD1KO macrophages than others. The high level of DCF fluorescence in the SOD1-deficient macrophages indicates that ROS levels are sustained in these cells, which is consistent with the report [7]. It is noteworthy that, upon LPS treatment, ROS levels were similar between DKO and SOD1KO macrophages, but viability was more extensively decreased in the case of DKO macrophages as seen in Fig. 1B–C. This suggests that NO produced by the SOD1KO macrophages, but absence in DKO macrophages, is involved in the rescue of the LPS-treated macrophages.

### 3.4 Cell survival is prolonged by a NO donor and aggravated by a NOS inhibitor in LPS-stimulated SOD1KO macrophages

**Fig. 5:**
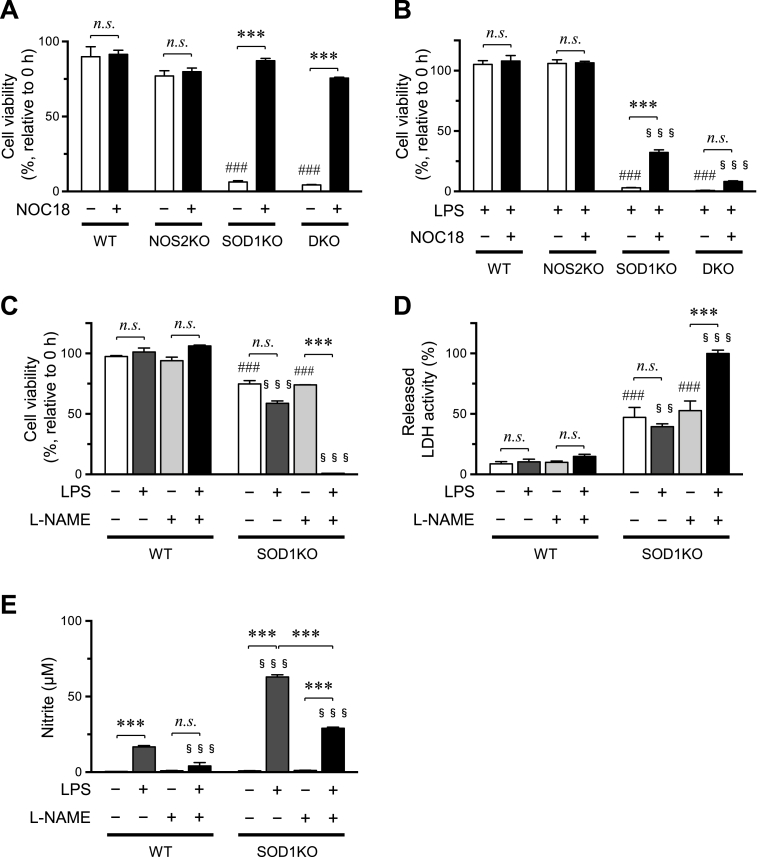
Verification of the protective effect of NO by means of a NO donor and a NOS2 inhibitor. (A) Macrophages were isolated from all four genotypic mice and treated with or without NOC18 (100 μM) for 96 h, and their numbers were counted. (B) At 0 h, macrophages were LPS treated (1.0 μg/ml) with or without NOC18 (100 μM) for 72 h. Because NOC18 is unstable, culture media in all samples were exchanged with fresh media containing indicated reagents every 24 h (A and B). Percentage of values to those at time 0 h are shown. Macrophages from WT and SOD1KO mice were incubated with LPS (1.0 μg/ml) and/or l-NAME (1.0 mM) for 72 h. The cellular viability (C) and released LDH activities (relative values to those of LPS-treated SOD1KO macrophages with l-NAME as 100% at time 72 h) (D) were measured at 72 h. Because nitrite is further oxidized to nitrate, levels of medium nitrite were measured at time 24 h (E). Data represent the mean ± SEM (n = 4). ***, P < 0.001; ###, P < 0.001 vs WT LPS (−); §§, P < 0.01 vs WT LPS (+); §§§, P < 0.001 vs WT LPS (+). (Tukey's test) n.s., not significant.

We examined the effect of NOC18, a NO donor that releases NO. Because the half-life of NOC18 is 21 h at pH7.4, culture media containing NOC18 and LPS were exchanged with fresh media every 24 h. The results indicated that the incubation of macrophage with NOC18 (100 μM, final concentration) actually improved the viability of SOD1KO and DKO macrophages, while it had no effect on the viability of WT or NOS2KO macrophages (Fig. 5A, Supplementary Fig. 1A). While NOC18 also improved the survival of SOD1KO macrophages that had been treated with LPS, it did not significantly improve the viability of the DKO macrophages (Fig. 5B). The reason for why NOC18 could not rescue the LPS-treated DKO macrophages can be attributed to a deficiency of intrinsic NO production by NOS2 as outlined in the Discussion section.

We then examined the effects of l-NAME, a specific inhibitor of NOS2 activity, in LPS-stimulated macrophages. When macrophages were treated with LPS, l-NAME (1.0 mM) was also included in the culture media and the media incubated for 72 h. As a result, while l-NAME alone had no effect on viability in either WT or SOD1KO macrophages, a marked aggravation in viability was observed on LPS stimulation (Fig. 5C, Supplementary Fig. 1B) and a simultaneous release of LDH (Fig. 5D) were observed only in the LPS-treated SOD1KO macrophages. Assaying the medium for nitrite content further confirmed the inhibition of NOS2 activity, although it was only partial in the SOD1KO macrophages (Fig. 5E) probably due to the exaggerated activation of NOS2. The viability of DKO macrophages was less than that of SOD1KO macrophages even under control conditions, (Fig. 1B). It is not surprising to see no difference in the viability of the SOD1KO macrophages by l-NAME treatment because NOS2 was not induced under control conditions. However, we do not know the reason why DKO macrophages was more vulnerable compared to the SOD1KO macrophage. This is the issue to be clarified in future study.

## 4 Discussion

The results reported herein provide support for the conclusion that NO produced by induced NOS2 under inflammatory stimuli exerts protective effects on SOD1-deficient macrophages most likely via scavenging superoxide. Upon LPS stimuli, the viability of DKO macrophages was decreased compared to those from SOD1KO mice (Fig. 1). NOC18, an NO donor, markedly ameliorated the viability of both SOD1KO and DKO macrophages under conventional culture conditions but also SOD1KO macrophages under stimulation by LPS (Fig. 4, Fig. 5A–B). On the contrary, the inhibition of NOS2 by l-NAME consistently deteriorated the viability of SOD1KO macrophages (Fig. 4, Fig. 5D). These data support the conclusion that NO, derived from either NOS2 or the NO donor, actually protected the cells against the cytotoxic action of superoxide with or without LPS stimulation. The protective action of NO can be attributed to decreasing superoxide levels, despite the production of peroxynitrite via interaction with superoxide. Although no reliable methods for measuring peroxynitrite are available, the superoxide-scavenging action of NO, which is robustly supported by in vitro-chemical analyses [11], provides a rationalization for the rescuing action of SOD1-deficient macrophages.

Chemical analysis data indicate that NO reacts very rapidly with superoxide (k = ~10^10^ M^−1^s^−1^), a rate that is about one-order more efficient than the SOD1-catalyzed dismutation of superoxide (k = ~1–2 x 10^9^ M^−1^s^−1^) [11]. Considering the cellular concentrations of NO produced by NOS2 and the SOD1 proteins, peroxynitrite would be clearly produced from NO and superoxide, even in the presence of SOD1 in cells. From a different perspective, this means that NO scavenges superoxide more efficiently than the SOD1-catalyzed reaction. Consistent with this conclusion, higher levels of superoxide and hydrogen peroxide were actually observed in alveolar macrophages from NOS2KO mice compared to WT macrophages [27]. In fact, the oxidation of low-density lipoprotein occurs more extensively by NOS2KO macrophages than WT macrophages upon stimulation with interferon-γ, and this accelerated oxidation is suppressed by a NO donor [28].

The LPS treatment stimulated NO production, as evidenced by accumulation of medium nitrite, in the WT and SOD1KO macrophages (Fig. 3A), which was consistent with the production of the NOS2 protein induced by the LPS treatment (Fig. 2). While ornithine levels were increased in all macrophages by the LPS treatment (Fig. 3C), arginine levels, though not significant, tended to increase in the SOD1KO macrophages (Fig. 3B). A robust increase in citrulline levels in the SOD1KO macrophages by the LPS treatment (Fig. 3D) strongly suggest that the conversion of arginine to citrulline is accelerated, which was consistent with the elevated nitrite levels (Fig. 3A). These observations were also consistent with the stimulated production of NO in SOD1 KO macrophages compared to WT macrophages. We previously reported that arginine uptake is stimulated by an LPS treatment in cultured mouse macrophages [18]. It has been also reported that the cationic amino acid transporter (CAT)-2, which is responsible for taking up arginine, is induced in stimulated macrophages [29]. Peroxynitrite reportedly induces the expression of CAT-2 but not CAT-1 in mesangial cells in the rat kidney [30]. Because superoxide production is elevated in many cells under inflammatory conditions and LPS treatment [1], NOS2, in coordination with induced CAT-2, produces more NO, which may be responsible for the suppression of the toxic action of superoxide, notably under conditions of an SOD1 deficiency.

NO derived from the NO donor largely rescued the viabilities of both SOD1KO and DKO macrophages without LPS treatment (Fig. 5A) but executed partial rescuing action on SOD1KO macrophages only upon LPS treatment (Fig. 5B). Because NOS2 was induced in SOD1KO cells by LPS stimulation, endogenously produced NO and NOC18-derived NO appeared to protect the SOD1KO cells from superoxide toxicity in a coordinated manner. The production of NO from NOS2 can be sustained for long periods of time, but the release of NO from NOC18 declines rapidly. Thus, in addition to the amounts of NO, differences in the sustainability of NO production might have differential effects on the viabilities of SOD1KO cells and DKO cells. It is conceivable that the SOD1-deficiency alone causes oxidative stress in macrophages under hyperoxygen conditions in culture compared to an in vivo situation. SOD1KO mice are phenotypically modest compared to cultivated cells, probably because, in addition to a relatively hypoxic in vivo situation, a variety of antioxidants, such as glutathione and ascorbic acid, are present in abundant levels, thus providing protection against oxidative damage in vivo. While no reliable method is currently available for measurement of superoxide in cells, superoxide levels in erythrocytes of SOD1KO mouse are calculated to be 200-times higher compared to those of WT mice [31]. This calculation appeared, at least partly, to be applicable to macrophages. Accordingly, it is conceivable that endogenous NO produced by activated NOS2 actually has a protective function against superoxide toxicity but that it is not produced in sufficient amounts for scavenging the majority of the elevated superoxide levels in the SOD1KO macrophages under LPS stimulation.

Peroxynitrite is a strong oxidant and reportedly exerts deteriorating effects on cells [11]. The overexpression of SOD1 protects RAW264.7 macrophages from apoptosis that is caused by both endogenous NO produced by NOS2 and exogenous NO released from the donor compound [32], which suggests that superoxide is actually involved in NO-induced cell death, most likely via the formation of peroxynitrite. In our study, superoxide reached toxic levels by itself due to a SOD1 deficiency and damaged the macrophages, eventually causing their death (Fig. 1B), most likely by impairing the signal function. Many cells contain Gpx1 and Prx member Prx2, both of which reportedly function as peroxynitrite reductase enzymes [25,26]. The rate constant for the catalysis by human Prx2 is moderate, 1.4 × 10^7^ M^−1^ s^−1^ at 25 °C and pH 7.4 [33], but substantial levels of these reductases may result in the reduction in peroxynitrite levels to allowable levels, which might consequently render cells resistant to the toxic action of the resulting peroxynitrite. The levels of these peroyxnitrite-reducing enzymes, Prx1-4 and Gpx1, were slightly different among macrophage groups or were changed differentially upon the LPS treatment (Fig. 2). The presence of these peroxynitrite-scavenging enzymes might contribute to the suppression of cytotoxicity of peroxynitrite and dominate the protective action of NO by means of scavenging superoxide, notably in SOD1KO cells.

There appeared to be discrepancy between cell viability as assessed by the release of LDH (Fig. 1C) and ROS levels (Fig. 4). The fluorescent probe H_2_DCF-DA, which is commonly used in detecting ROS, actually reacts with several different peroxides including peroxynitrite and possibly other reactive species as well, but does not effectively detect superoxide [34]. It is, therefore, not surprising to see the inconsistent results between viability and ROS levels, as assessed by means of H_2_DCF-DA because NO mainly scavenges superoxide. We were not able to specifically detect superoxide in the cells because superoxide is rather labile and there is no superoxide-specific probe currently available. It is generally understood that the oxidizing power of superoxide itself is not so strong compared to other ROS such as hydroxyl radicals or peroxynitrite. However, transferring the unpaired electron of superoxide to another molecules may result in the production of more toxic radical species, notably hydroxyl radicals by means of the iron-mediated Fenton reaction [35]. Consequently, a radical chain reaction initiated by a radical electron from superoxide could be involved in the enhancement in the lipid peroxidation reaction [36]. A recent study reported that lipid peroxidation products are typically produced in the case of a defect in the glutathione-glutathione peroxidase 4 axis, trigger iron-dependent, non-apoptotic cell death, referred to as ferroptosis [37]. Because SOD suppresses radical chain reactions by converting superoxide to hydrogen peroxide at the initial step [6], the elimination of superoxide radicals by NO would also suppress the formation of lipid peroxides and consequently allow ferroptotic cell death to be avoided. As the nature of the interaction of these reactive molecules in vivo remain a complex issue, further studies will clearly be required to completely understand the actual physiological meanings of the interaction between NO and superoxide.

In conclusion, the data presented in this study indicate the NO exerts a cytoprotective action under conditions where an excessive amount of superoxide is present due to a SOD1 deficiency. Despite the production of peroxynitrite, the viability of the SOD1KO macrophages was markedly ameliorated by NO most likely via its superoxide-scavenging function. These results further imply that, in addition to its well-established signaling function, NO plays crucial roles in antioxidation and assists in coping with the cytocidal effects of superoxide.

## Author contributions

SK and TH equally contributed the experimental data. JF arranged the experiments and wrote the manuscript.

## Funding

This work was supported by the 10.13039/501100001691JSPS KAKENHI Grant-in-Aid for Scientific Research (C) (18K06948) to JF from the 10.13039/501100001691Japan Society for the Promotion and Science (JSPS) and, in part, by the YU-COE program [M30-3] and [C31-3] to SK, TH, and JF from Yamagata University.

## Declaration of competing interest

The authors declared no conflicts of interest.
