# Supplementary material for: Nitric oxide produced by NOS2 copes with the cytotoxic effects of superoxide in macrophages
Source: Biochem Biophys Rep. 2021 Feb 20;26:100942. doi: 10.1016/j.bbrep.2021.100942 (PMC7905073; doi:10.1016/j.bbrep.2021.100942)
Supplement: Multimedia component 1 [file mmc1.docx]

**Supplementary Table 1.** After measurement of amino acids and related compounds in the cells by LC-MS, values that show difference among mouse groups are indicated. Data are expressed as nmol/mg protein and represent mean ± SEM (n=3). *, P<0.05; **, P<0.01; ***, P<0.001 vs control of each genotype; ^#^, P<0.05; ^##^, P<0.01; ^###^, P < 0.001 vs WT control; ^§^, P<0.05; ^§§^, P<0.01; ^§§§^, P < 0.001 vs WT LPS (+). (Tukey's test)

|  | WT | | NOS2KO | | SOD1KO | | DKO | |
| --- | --- | --- | --- | --- | --- | --- | --- | --- |
|  | cont | LPS | cont | LPS | cont | LPS | cont | LPS |
| Cysteine | 1.7 ± 0.1 | 4.0 ± 0.4^***^ | 1.2 ± 0.2 | 2.7 ± 0.2^**,§^ | 2.3 ± 0.3 | 3.8 ± 0.1^**^ | 1.4 ± 0.1 | 1.5 ± 0.2^§§§^ |
| Phenylalanine | 0.76 ± 0.13 | 1.21 ± 0.05 | 0.69 ± 0.03 | 0.73 ± 0.07 | 0.75 ± 0.14 | 1.52 ± 0.21^**^ | 0.89 ± 0.11 | 1.40 ± 0.12 |
| Leucine | 2.8 ± 0.5 | 4.3 ± 0.1 | 2.7 ± 0.1 | 3.6 ± 0.3 | 3.0 ± 0.3 | 6.8 ± 0.1^**,§^ | 3.7 ± 0.7 | 7.1 ± 1.0^**,§^ |
| Isoleucine | 2.5 ± 0.3 | 3.7 ± 0.1 | 2.4 ± 0.2 | 3.1 ± 0.4 | 2.7 ± 0.5 | 6.2 ± 0.6^**,§^ | 4.1 ± 0.7 | 7.1 ± 0.6^**,§§^ |
| Methionine | 0.62 ± 0.15 | 0.80 ± 0.04 | 0.48 ± 0.07 | 0.61 ± 0.19 | 0.38 ± 0.12 | 1.13 ± 0.01 ^*^ | 0.41 ± 0.19 | 0.29 ± 0.11 |
| Glutathione (reduced) | 19 ± 1 | 13 ± 1 | 22 ± 2 | 12 ± 1^*^ | 26 ± 1 | 25 ± 1^§§^ | 34 ± 1^###^ | 30 ± 4^§§§^ |
| Proline | 0.34 ± 0.09 | 0.50 ± 0.03 | 0.28 ± 0.01 | 0.51 ± 0.11 | 0.46 ± 0.15 | 1.21 ± 0.31^*,§^ | 0.47 ± 0.10 | 1.04 ± 0.12 |
| Carnitine | 1.15 ± 0.02 | 0.44 ± 0.04^***^ | 0.98 ± 0.06^#^ | 0.40 ± 0.04^***^ | 0.52 ± 0.05^###^ | 0.15 ± 0.01^***,§§§^ | 0.51 ± 0.02^###^ | 0.20 ± 0.01^***,§§^ |
| Acetyl choline | 0.148 ± 0.001 | 0.119 ± 0.009 | 0.110 ± 0.008^#^ | 0.105 ± 0.006 | 0.075 ± 0.009^###^ | 0.082 ± 0.003^§^ | 0.085 ± 0.002^###^ | 0.091 ± 0.008 |
| Sarcosine | 0.105 ± 0.002 | 0.123 ± 0.003 | 0.124 ± 0.009 | 0.113 ± 0.001 | 0.149 ± 0.008 | 0.158 ± 0.007 | 0.213 ± 0.014^##^ | 0.410 ± 0.036^***,§§§^ |
| Taurine | 107 ± 1 | 66 ± 3 | 159 ± 31 | 52 ± 4^*^ | 121 ± 8 | 113 ± 13 | 193 ± 38 | 69 ± 14^**^ |
| Asparagine | 2.9 ± 0.2 | 4.9 ± 0.1 | 2.6 ± 0.2 | 6.0 ± 0.3^**^ | 4.3 ± 0.7 | 7.2 ± 0.3^*^ | 5.1 ± 0.8 | 8.8 ± 0.9^**,§§^ |
| Glutamine | 6.2 ± 0.5 | 9.2 ± 0.3 | 6.8 ± 0.4 | 11.2 ± 0.7 | 8.3 ± 0.9 | 13.9 ± 1.1 | 11.7 ± 1.8 | 17.8 ± 2.0^*,§§^ |
| γ-aminobutyric acid | 3.40 ± 0.06 | 1.47 ± 0.13^***^ | 2.59 ± 0.09^#^ | 1.01 ± 0.02^***^ | 1.97 ± 0.21^###^ | 0.97 ± 0.02^**^ | 2.16 ± 0.19^###^ | 1.70 ± 0.30 |
| β-alanine | 31.9 ± 0.9 | 12.0 ± 0.6^***^ | 27.0 ± 1.7 | 7.1 ± 0.1^***^ | 23.3 ± 2.6^##^ | 14.9 ± 0.9^**^ | 22.7 ± 1.0^##^ | 7.1± 1.1^***^ |
| Carnosine | 0.042 ± 0.003 | 0.062 ± 0.003^*^ | 0.029 ± 0.002 | 0.035 ± 0.001^§§§^ | 0.059 ± 0.003^#^ | 0.093 ± 0.001^***, §§§^ | 0.069 ± 0.003^###^ | 0.098 ± 0.007^***,§§§^ |
| 2-aminoadipic acid | 0.13 ± 0.01 | 0.19 ± 0.01 | 0.15 ± 0.01 | 0.18 ± 0.01 | 0.17 ± 0.02 | 0.25 ± 0.02^*^ | 0.23 ± 0.01^##^ | 0.23 ± 0.02 |

**Supplementary Table 1** Continued

|  | WT | | NOS2KO | | SOD1KO | | DKO | |
| --- | --- | --- | --- | --- | --- | --- | --- | --- |
|  | cont | LPS | cont | LPS | cont | LPS | cont | LPS |
| Aspartic acid | 8.7 ± 0.8 | 14.9 ± 1.1^*^ | 6.7 ± 0.3 | 17.3 ± 1.3^***^ | 7.7 ± 1.2 | 15.0 ± 2.1^**^ | 7.4 ± 0.8 | 8.9 ± 0.6^§^ |
| o-phosphoethanolamine | 11.8 ± 0.4 | 23.0 ± 1.2^***^ | 9.8 ± 0.8 | 10.2 ± 0.7^§§§^ | 10.9 ± 0.6 | 32.0 ± 1.9^***,§§§^ | 10.1 ± 0.3 | 13.6 ± 1.3^§§§^ |
| Choline | 0.80 ± 0.04 | 1.15 ± 0.10 | 0.72 ± 0.11 | 2.02 ± 0.16^***,§§§^ | 0.69 ± 0.07 | 1.01 ± 0.05 | 0.79 ± 0.13 | 1.00 ± 0.11 |
| Lysine | 0.86 ± 0.14 | 2.70 ± 0.04^***^ | 0.88 ± 0.09 | 1.91 ± 0.09^*^ | 1.00 ± 0.17 | 3.55 ± 0.18^***^ | 1.49 ± 0.26 | 4.27 ± 0.35^***,§§§^ |
| γ-glutamyl-cysteine | 0.084 ± 0.003 | 0.189 ± 0.019^**^ | 0.129 ± 0.007 | 0.060 ± 0.003^§§§^ | 0.113 ± 0.005 | 0.208 ± 0.014^**^ | 0.181 ± 0.023^##^ | 0.247 ± 0.027 |
| γ-glutamyl-alanine | 0.270 ± 0.011 | 0.011 ± 0.001^***^ | 0.272 ± 0.015 | 0.0082 ± 0.0002^***^ | 0.125 ± 0.014^###^ | 0.026 ± 0.003^***^ | 0.148 ± 0.006^###^ | 0.019 ± 0.009^***^ |
