# Supplementary material for: Nitric oxide produced by NOS2 copes with the cytotoxic effects of superoxide in macrophages
Source: Biochem Biophys Rep. 2021 Feb 20;26:100942. doi: 10.1016/j.bbrep.2021.100942 (PMC7905073; doi:10.1016/j.bbrep.2021.100942)
Supplement: Multimedia component 2 [file mmc2.pptx]

## Slide 1
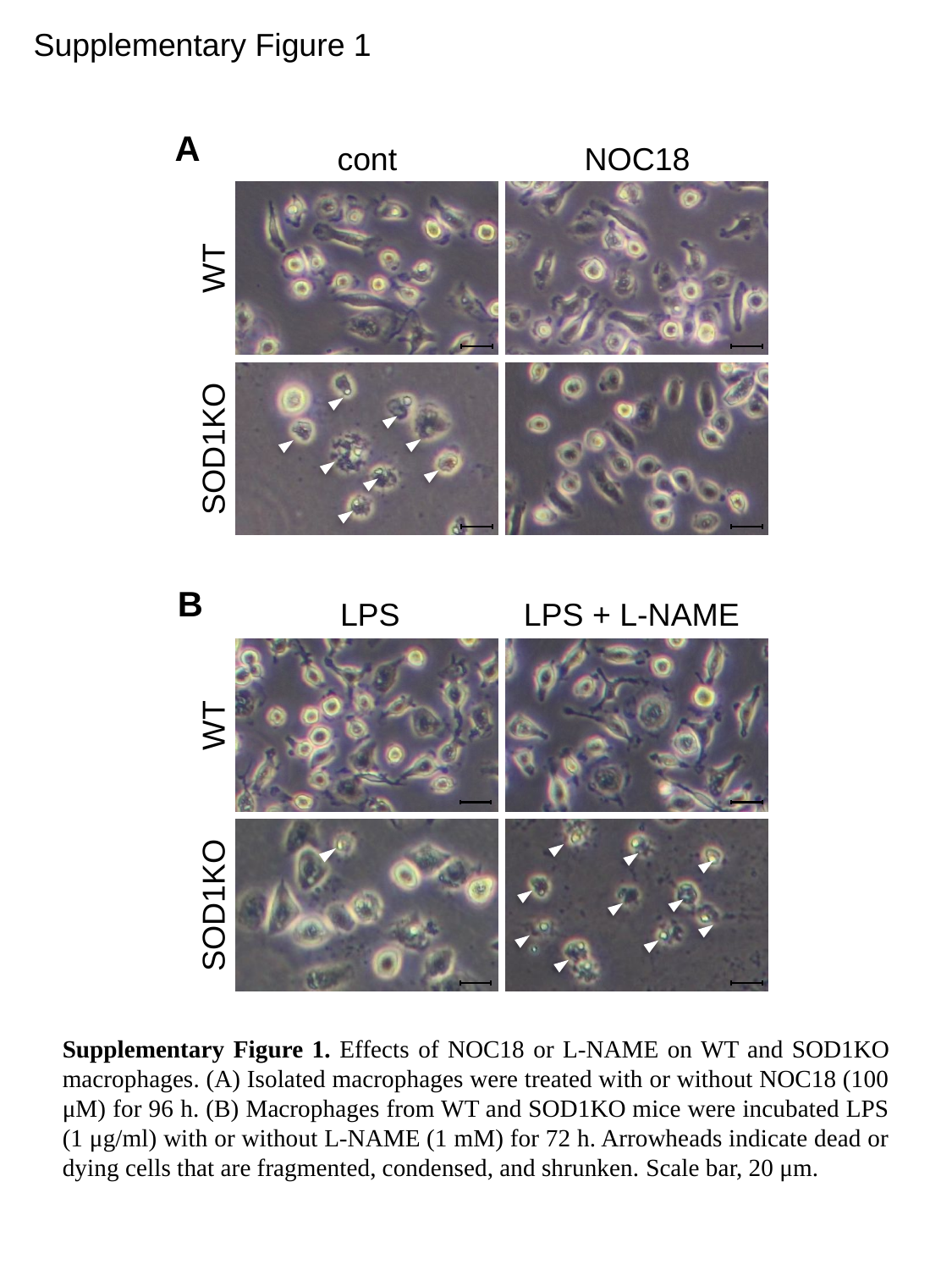

Supplementary Figure 1
A
cont
NOC18
WT
SOD1KO
B
LPS
LPS + L-NAME
WT
SOD1KO
Supplementary Figure 1. Effects of NOC18 or L-NAME on WT and SOD1KO macrophages. (A) Isolated macrophages were treated with or without NOC18 (100 μM) for 96 h. (B) Macrophages from WT and SOD1KO mice were incubated LPS (1 μg/ml) with or without L-NAME (1 mM) for 72 h. Arrowheads indicate dead or dying cells that are fragmented, condensed, and shrunken. Scale bar, 20 μm.
